# Ten fraction hypofractionated stereotactic body radiotherapy for the management of ultracentral lung tumors: a retrospective analysis of dosimetry, outcomes, and toxicity

**DOI:** 10.1186/s13014-023-02298-1

**Published:** 2023-08-02

**Authors:** Crosby Rock, Sumit Sood, Ying Cao, Shary Shelton, Ronald C. Chen, Fen Wang

**Affiliations:** 1 Department of Radiation Oncology, University of Kansas Medical Center, Kansas City, KS USA; 2 Department of Radiation Oncology, University of Minnesota, Minneapolis, MN USA

**Keywords:** Ultracentral, Hypofractionated, Stereotactic body radiotherapy, Lung

## Abstract

**Background:**

The management of ultracentral thoracic tumors with ablative dose of radiotherapy remains challenging given proximity to critical central structures. We report patient outcomes, toxicity, and dosimetry for ultracentrally located tumors with hypofractionated stereotactic body radiotherapy (hfSBRT).

**Methods:**

Seventy-eight individuals (50 initial radiotherapy, 28 re-irradiation) undergoing 10 fraction hfSBRT for ultracentrally located thoracic tumors treated between 2009 and 2020 at a single institution were retrospectively reviewed. Overall survival (OS), progression free survival (PFS), and local control (LC) were calculated. Incidence and grade of treatment related toxicity were evaluated. Dosimetric analysis of treatment plans and critical adjacent OARs was performed.

**Results:**

At a median follow up time of 13 months, 1- and 3-year OS, PFS, and LC were 89%/63%, 37%/18%, and 84%/65%, respectively. Median dose was 65 Gy (BED_10_ = 107.25 Gy). Median primary bronchial tree maximum dose (Dmax) was 60 Gy (V50 = 0.96 cc). Median esophageal Dmax was 38 Gy (V40 = 0 cc). Median great vessel Dmax was 68 Gy (V50 = 3.53 cc). The most common ≥ grade 2 adverse event was pneumonitis, in 15 individuals (20%). Grade 3 or higher toxicity was observed in 9 individuals (12%): three cases of grade 3 pneumonitis (two re-irradiation, one initial radiotherapy), one grade 3 esophageal stricture following re-irradiation, two grade 3 endobronchial obstructions both following initial radiotherapy, and three grade 5 hemoptysis events (two re-irradiation, one initial radiotherapy). One hemoptysis event was categorized as “possibly” related to treatment, while the remaining two events were categorized as “unlikely” related to treatment in patients with clear evidence of disease progression.

**Conclusions:**

hfSBRT to ultracentral lung tumors delivered over 10 fractions is a safe and effective treatment option, with acceptable rates of toxicity and good rates of tumor control.

*Trial registration*: IRB registration number 12573.

## Background

Stereotactic body radiotherapy (SBRT) continues to play an expanding role in the management of primary lung cancer and that of intrathoracic metastatic disease. SBRT has been established as the standard of care in the management of medically inoperable early-stage non-small cell lung cancer (NSCLC) [1, 2], with emerging data to support its efficacy and minimal toxicity for those otherwise eligible for surgical intervention [3–5]. Furthermore, SBRT in the management intrathoracic oligoprogressive or oligometastatic disease, has demonstrated promising results [6, 7]. Initial SBRT trials investigating the feasibility and safety of SBRT raised concerns in the management of centrally located tumors suggesting an increased risk of severe toxicity [8]. This gave rise to the term “No Fly Zone,” referring to tumors located within 2 cm in all directions from the proximal bronchial tree. To reduce the incidence of severe toxicity in centrally located tumors, risk adapted dose fractionation schemes were developed, maintaining excellent oncologic outcomes with acceptable toxicity profiles [9, 10].

Given the narrow therapeutic ratio of SBRT for centrally located tumors, especially when considering tumors directly abutting or involving critical mediastinal structures, the term “ultracentral” lung tumor has arisen. Multiple treatment strategies have been reported; however, adoption of a standardized treatment regimen has not been established. Reasons for this include variable definitions of an ultracentral tumor, as well as the heterogenicity of dose and fractionation schedules reported in the literature. Individuals have undergone treatment in as few as three and as many as ≥ 10 fractions, corresponding to biologic effective doses (BED_10_) of 48 Gy to over 100 Gy [11–13]. As a result, establishing consistent practice guidelines and evaluation of treatment efficacy for ultracentral lung tumors has been difficult.

The current retrospective study aims to establish the feasibility of a hfSBRT (10-fraction) regimen by characterizing its safety and efficacy in both the upfront setting for primary and metastatic lung tumors, as well as in recurrent disease requiring re-irradiation for tumors located in an ultracentral location. The basis for adoption of a 10-fraction treatment regimen at our institution was largely due to early published works demonstrating an increased safety profile when utilizing a protracted treatment course for both centrally and ultracentrally located tumors when compared to initial SBRT studies [9, 10, 14, 15].

## Methods

### Patient population

This retrospective analysis includes 78 individuals (50 primary and 28 reirradiation) treated from 2009 to 2020 who underwent hfSBRT with 10 fractions for ultracentrally located lung tumors at the University of Kansas Medical Center. Patients with primary (n = 33) or recurrent lung cancer (n = 29) as well as those with oligometastatic intrathoracic disease (n = 16) were included in this Institutional Review Board-approved retrospective study.

Selection criteria to receive hfSBRT include: (1) Curative intent treatment in the setting of primary or recurrent disease, or local ablation in oligometastatic disease. (2) No concurrent systemic therapy. (3) Ultracentral location defined as PTV overlap or direct tumor abutment with the major vessels (aorta, pulmonary artery, pulmonary veins, and superior and inferior vena cava), esophagus, or central airway. Patients are classified as receiving re-irradiation if the PTV overlapped with the 50% isodose line (IDL) of prior treatment plan.

### hfSBRT treatment

Patients were simulated on a Philips CT simulator (Amsterdam, Netherlands) using abdominal compression and individually shaped body fixation devices (BlueBAG, BodyFIX system, Medical Intelligence, Schwabmuenchen, Germany). Treatment volumes were derived utilizing a 4-dimensional (4D) computed tomography (CT) simulation scan. Generation of an internal target volume (ITV) was delineated on maximum intensity projection (MaxIP) image sets. A uniform 5 mm ITV expansion was utilized for the construction of the PTV. Organs-at-risk (OARs), including the bilateral lungs, heart, spinal cord, esophagus, major vessels, and primary bronchus, were contoured on mean intensity projection image sets. All patients underwent 10-fraction hfSBRT and were treated daily or every other day. Total treatment dose was determined by the treating physicians, and dose reduction in instances of unacceptably high OAR doses were permitted. Institutional guidelines for OARs were a point maximal dose (Dmax) of 32 Gy to the spinal canal, a Dmax of 48 Gy and V40 (percentage volume receiving at least 40 Gy) < 5 cc for the esophagus, and Dmax of 42 Gy for the brachial plexus. PTV underdosage was allowed to avoid exceeding these limits. There were no specific dose limits for the lung, heart, trachea, and main bronchi, but with intent to maximally reduce dose to these OARs.

Treatments plans were generated utilizing either 3D (iPlan, Brain lab, Munich, Germany) conformal or IMRT/VMAT (Eclipse, Varian, Palo Alto, California) techniques and were optimized to achieve 95% or higher of PTV volume receiving 100% of the prescription dose. Treatment doses ranged from 40 to 70 Gy in 10 fractions corresponding to a biologically effective dose of 56–119 Gy (α/β  = 10, BED_10_).

For individuals undergoing re-irradiation, prior treatment records carefully reviewed to estimate treatment volume overlap with prior treatment fields, and to estimate the cumulative dose to OARs.

### Clinical endpoints

Local tumor control rate (LC), progression free survival rate (PFS), overall survival rate (OS) were calculated from the date of treatment completion. hfSBRT related toxicities were assessed. Post-treatment follow up was performed every 3 to 6 months including a CT scan of the thorax. Treatment failure was determined utilizing CT radiographic evidence. If disease progression was suspected, patients underwent positron emission tomography (PET). In instances of equivocal CT and PET results, a minority of patients underwent tissue biopsy. Toxicity was categorized according to the Common Terminology Criteria of Adverse Events (CTCAE) v 5.0.

### Dosimetric analysis

Dosimetric analysis of OARs was undertaken. The primary bronchial tree was delineated by contouring the lumen of the entire main bronchus in both mediastinal and lung settings. The same procedure was performed for the tracheal wall, which was contoured 2 cm craniocaudally to the PTV. Similarly, the great vessels and esophagus were contoured 2 cm craniocaudally to the PTV and expanded using at least two phases of the 4D CT. Lung contours were automatically generated with subtraction of the PTV. Maximal doses (Dmax) for the esophagus, bronchial tree, and great vessels were reported. Further dosimetric variables including esophageal V40, bronchial V50, and great vessel V50 were included. Bilateral lung V5, V20, V40 and mean lung dose (MLD) were described.

For individuals undergoing thoracic re-irradiation prior radiation doses, radiation technique (EBRT or SBRT), and time interval between radiotherapy courses were reported.

### Statistical analysis

Descriptive statistics were used summarize cohort data including patient demographics, pretreatment risk factors, as well as tumor characteristics. Treatment outcomes including LC, PFS, and OS were estimated using the Kaplan–Meier (KM) method. Statistical significance in clinical outcomes for primary, metastatic, and recurrent disease were assessed utilizing Fisher’s exact and Chi-square tests as well as the Kaplan–Meier method. Differences in treatment regimens, dosimetry, and adverse events among individuals undergoing first course vs re-irradiation was performed using the Fisher’s exact, Wilcoxon rank sum, and Chi-square tests. Univariate linear regression analysis was performed to evaluate associations between treatment outcomes (LC, PFS and OS) and patient, tumor, or dosimetric characteristics. A *p*-value of < 0.05 was considered significant for all measurements performed. All statistical analyses were performed using SAS 9.4 (SAS institute, Inc, Cary, North Carolina).

## Results

### Patient and tumor characteristics

In total 78 patients were included. Median age was 70.6 years (range, 38.8–96.7 years). Median follow up was 13.1 month and ranged from 0.3 to 102 months. Patients underwent hfSBRT for primary lung cancer, recurrent thoracic, and oligometastatic disease 42.3% (n = 33), 37.2% (n = 29) and 20.5% (n = 16) of the time, respectively. Twenty-eight of the 29 individuals (96.6%) undergoing hfSBRT for recurrent disease were treated in locations with PTV volume overlap with the 50% IDL of prior radiation therapy field (re-irradiation). Treated tumors were most commonly primary lung adenocarcinomas (n = 36, 46.1%). Tumors were in the hilum (n = 29, 37.2%), mediastinum (n = 22, 28.2%), mediastinum and hilum (n = 15, 19.2%), and lung parenchyma and hilum (n = 12, 15.4%) (Table 1).

**Table 1:** Detailed patient and tumor characteristics

Characteristic	N (%)
Sex	
Male	35 (45.9)
Female	43 (55.1)
Age (years): median (range)	70.6 (38.9–96.7)
Risk factors	
COPD	37 (47.4)
ECOG performance status: median (range)	2 (0 to 3)
FEV1: median % (range)	66.5 (23–112)
DLCO: median % (range)	60.5 (26–103)
Histology	
Adenocarcinoma	36 (46.2)
SCC	27 (34.6)
Other	14 (17.9)
No biopsy	1 (1.3)
Ultra-central tumor location	
Hilum	29 (37.2)
Mediastinum	22 (28.2)
Mediastinum + Hilum	15 (19.2)
Parenchyma + Hilum	12 (15.4)
Patient disease status prior to treatment	
Primary	33 (42.3)
Metastatic	16 (20.5)
Recurrent	29 (37.2)
PTV Volume (cc): median (range)	66.6 (9.3–1201.8)
PET Maximum SUV prior to treatment: Median (range)	8.9 (1.3–30.2)
Follow-up time (months): median (range)	13.1 (0.3–102.3)
Prior chemotherapy	
Yes	44 (56.4)
No	34 (43.6)
Thoracic re-irradiation treatment characteristics (n = 28)	
Previous thoracic radiation	
EBRT	17 (60.8)
SBRT	7 (25.0)
Both	4 (14.2)
Dose of previous thoracic radiation (Gy)	
EBRT: median (range)	66 (50.0–70.2)
SBRT: Median (range)	50 (19.5–70.0)
Time Interval from previous radiation (months): Median (range)	16.1 (3.6–100.9)

*COPD:* chronic obstructive pulmonary disease, *ECOG:* Eastern cooperative oncology group, *FEV1:* Forced expiratory volume in 1 s, *DLCO:* Diffusion capacity of the lung for carbon monoxide, *SCC:* squamous cell carcinoma, *PTV:* planning target volume, *PET:* positron emission tomography, *SUV:* standardized uptake value, *EBRT:* external beam radiation therapy, *SBRT:* stereotactic body radiation therapy

### Treatment outcomes

**Fig. 1:**
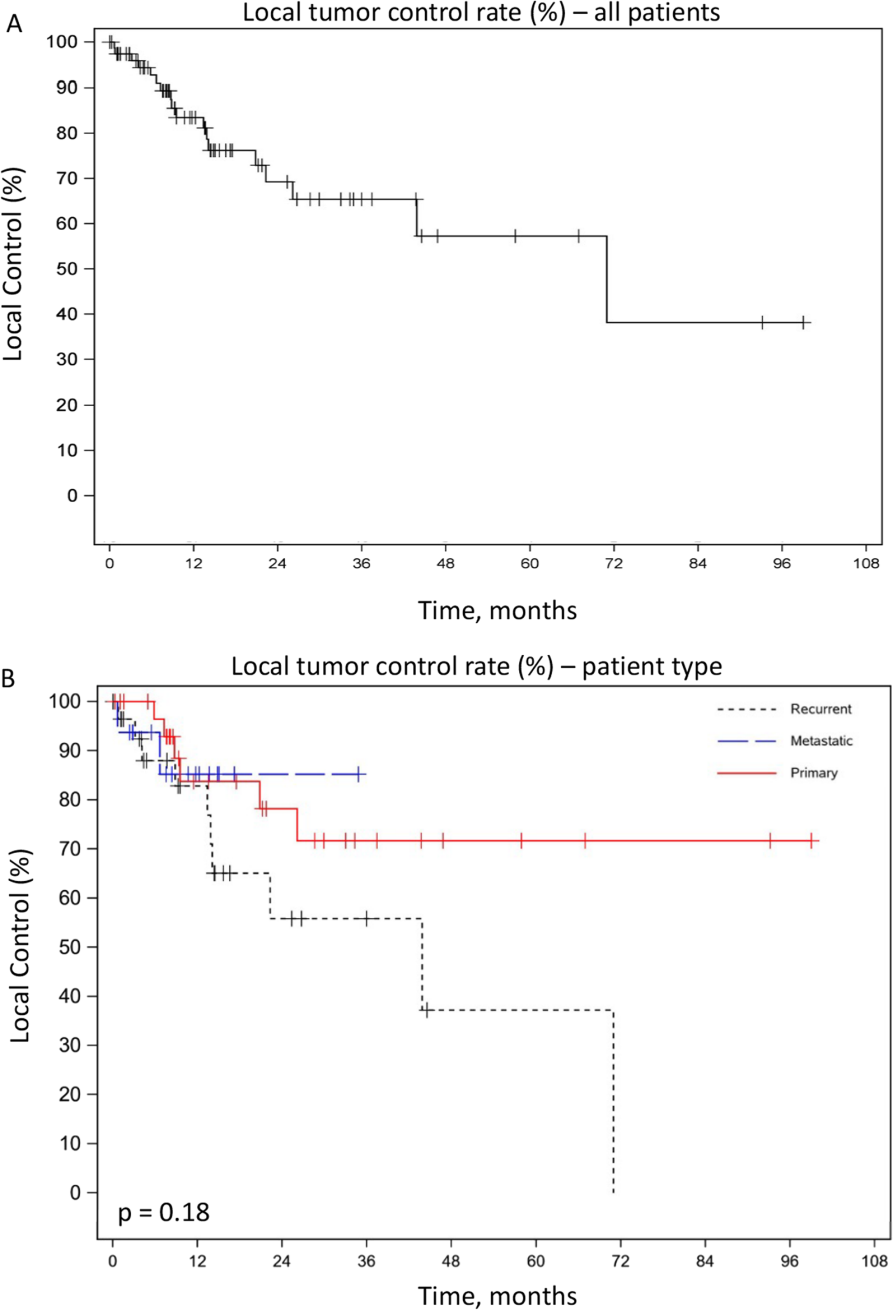
Kaplan–Meier Survival Curves—Local Control, **A** Local control rate of tumor as a percent plotted against time in months of all patients, combined. **B** Local control rate of tumor as a percent plotted against time in months of three categories of patients. The patient categories are primary, recurrent, or metastatic

Local control at 1- and 3-years are 83.5% and 65.4%, respectively (Fig. 1A). No difference was observed in 1- or 3-year LC rates in univariate subgroup analysis comparing primary (1-year LC = 83.8%, 3-year LC = 65.4%), recurrent (1-year LC = 83.8%, 3-year LC = 57.6%) or metastatic disease (1-year LC = 85.2%) (p = 0.99 and 0.12, respectively) (Table 2). Similarly, KM survival analysis comparing LC for primary, recurrent, or metastatic disease was not statistically different (*p* = 0.18) (Fig. 1B).

**Table 2:** Patient outcomes

	Entire cohort	Primary disease	Recurrent disease	Metastatic disease	*p*-value
*Overall survival*					
1-year rate, %	89.2	93.7	83.5	88.9	0.42
3-year rate, %	63.4	70.5	52.2	44.4	0.16
*Progression free survival*					
1-year rate, %	37.3	50.1	38	12.5	0.05
3-year rate, %	18.1	26.8	19	0	0.07
*Local control*					
1-year rate, %	83.5	83.8	82.8	85.2	0.99
3-year rate, %	65.4	71.7	57.6	–	0.12

**Fig. 2:**
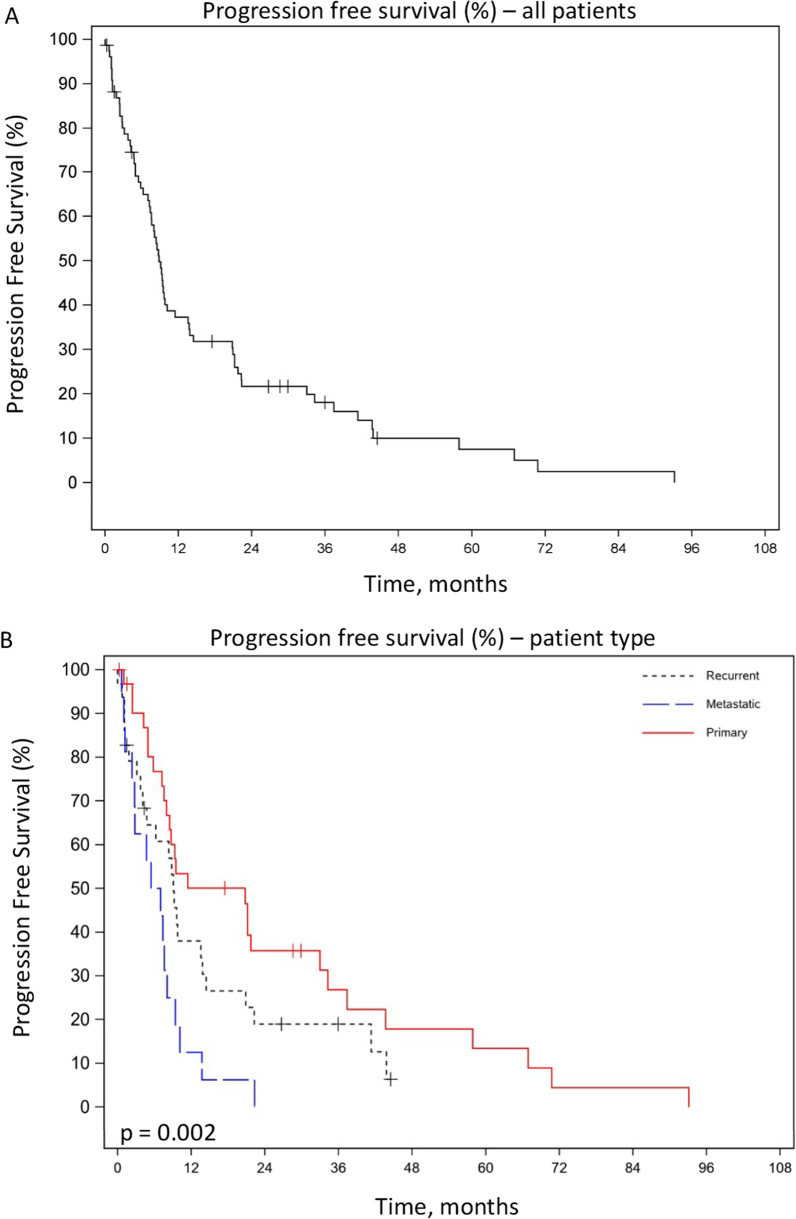
Kaplan–Meier Survival Curves—Progression Free Survival, **A** Progression free survival rate as a percent plotted against time in months of all patients, combined. **B** Progression free survival rate as a percent plotted against time in months of three categories of patients. The patient categories are primary, recurrent, or metastatic

Despite favorable local control, 1- and 3-year PFS rates were 37.3% and 18.1%, respectively (Fig. 2A). Trends toward significance were observed in both 1- and 3-year PFS rates comparing primary (1-year PFS = 50.1%, 3-year PFS = 26.8%), recurrent (1-year PFS = 38.0%, 3-year PFS = 19.0%), and metastatic disease (1-year PFS = 12.5%, 3-year PFS = 0%) (*p* = 0.05 and 0.07, respectively) (Table 2). Superior PFS was observed for those undergoing hfSBRT for primary disease on KM analysis (*p* = 0.002) (Fig. 2B).

**Fig. 3:**
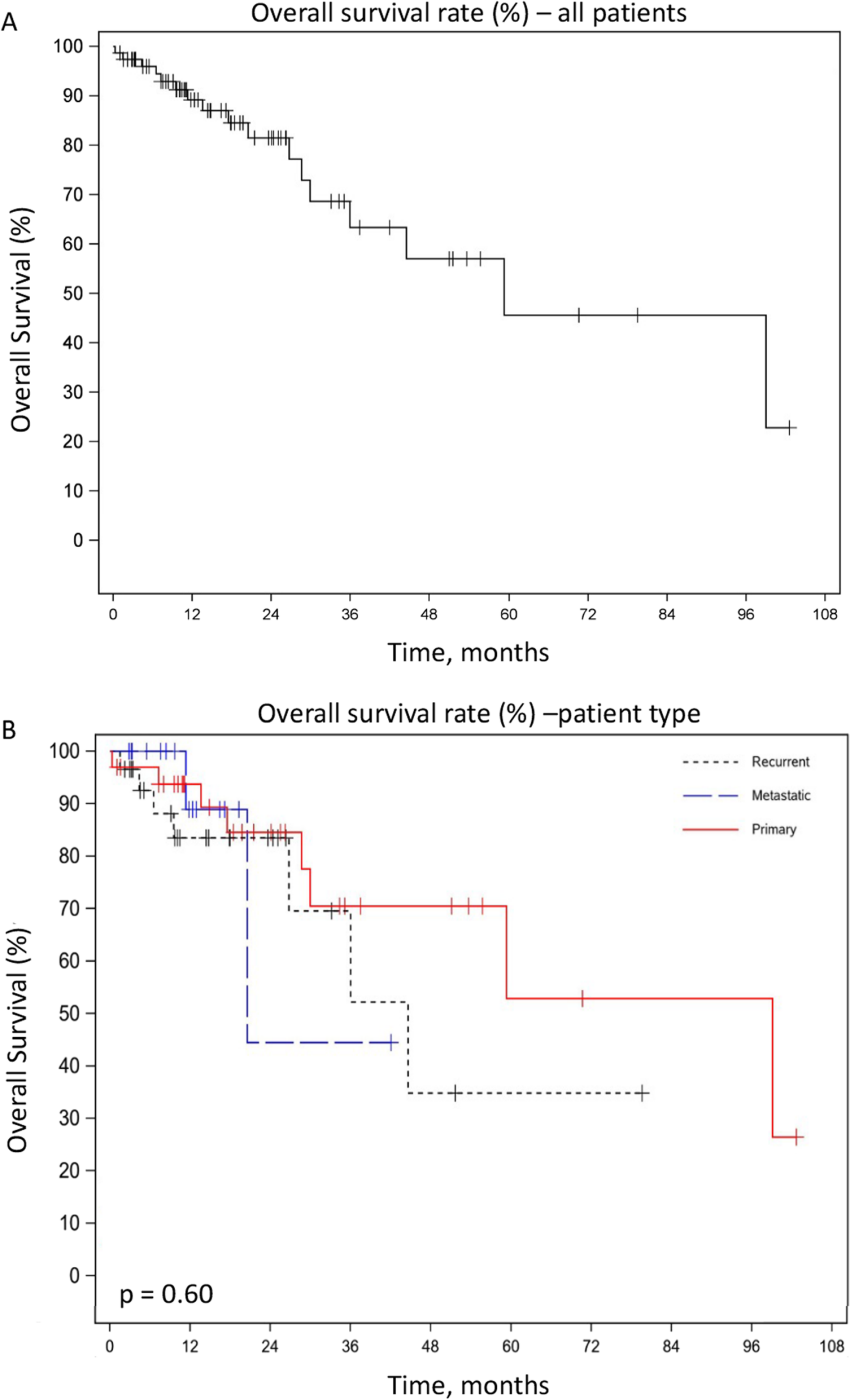
Kaplan–Meier Survival Curves—Progression Free Survival, **A** Overall survival rate as a percent plotted against time in months of all patients, combined. **B** Overall survival rate as a percent plotted against time in months of three categories of patients. The patient categories are primary, recurrent, or metastatic

One and 3-year OS rates for the entire patient cohort were 89.2% and 63.4%, respectively (Fig. 3A). On subgroup analysis, OS rates were: primary (1-year OS = 93.7%, 3-year OS = 70.5%), recurrent (1-year OS = 83.5%, 3-year OS = 52.2%) or metastatic disease (1-year OS = 88.9%, 3-year OS = 44.4%), although these differences were not statistically significant (*p* = 0.42 and 0.16, respectively) (Table 2 and Fig. 3B).

On univariate analysis no statistically significant correlates between treatment outcome (LC, PFS and OS) and patient or therapy characteristics were identified, though there was a trend toward inferior control in those undergoing re-irradiation (*p* = 0.07) (Table 3).

**Table 3:** Univariate logistic regression model: Factors associated with local control of tumor, progression free survival and overall survival

Characteristic	Local control		Progression free survival		Overall survival	
	OR (95% CI)	*p*-value	OR (95% CI)	*p*-value	OR (95% CI)	*p*-value
SBRT total dose	1.05 (0.98–1.19)	0.15	0.97 (0.90–1.05)	0.51	1.03 (0.97–1.10)	0.3
SBRT BED_10_	1.02 (0.99–1.05)	0.13	0.99 (0.95–1.03)	0.53	1.02 (0.97–1.05)	0.3
Metastatic or recurrent disease (reference = primary disease)	1.63 (0.54–4.93)	0.38	1.48 (0.39–5.61)	0.56	0.78 (0.27–2.30)	0.65
Re-irradiation (reference = no prior radiation)	2.70 (0.92–7.92)	0.07	0.56 (0.15–2.12)	0.39	1.24 (0.41–3.72)	0.7
Histology (reference = other)						
Adenocarcinoma	0.63 (0.01–62.45)	0.77	3.26 (0.03–337.09)	0.61	0.75 (0.01–73.55)	0.9
Squamous cell carcinoma	1.78 (0.02–173.35)	0.12	2.28 (0.02–238.09)	0.72	0.72 (0.01–72.60)	0.89
Tumor location (reference = lung parenchyma + hilum)						
Mediastinum	1.02 (0.19–6.70)	0.98	0.87 (0.15–5.23)	0.88	1.40 (0.29–6.69)	0.67
Hilum	1.66 (0.37–9.99)	0.55	1.80 (0.29–11.23)	0.52	0.36 (0.07–1.98)	0.24
Mediastinum + hilum	1.64 (0.29–11.14)	0.59	6.42 (0.25–167.81)	0.26	0.91 (0.15–5.42)	0.91
ITV	1.00 (0.97–1.01)	0.78	0.99 (0.99–1.00)	0.11	1.00 (0.99–1.00)	0.27
PTV	1.00 (0.99–1.00)	0.73	0.99 (0.99–1.00)	0.1	1.00 (0.99–1.00)	0.29
D98	1.03 (0.97–1.09)	0.28	0.98 (0.92–1.07)	0.74	1.03 (0.97–1.09)	0.34
D99	1.03 (0.98–1.09)	0.27	0.99 (0.91–1.07)	0.78	1.02 (0.97–1.09)	0.41
PET SUV max	1.00 (0.90–1.12)	0.98	0.99 (0.85–1.16)	0.91	0.94 (0.80–1.09)	0.4
Age	0.99 (0.94–1.03)	0.64	1.05 (0.99–1.12)	0.12	0.99 (0.92–1.02)	0.22
Sex (reference = female)	1.75 (0.61–5.06)	0.3	0.81 (0.21–3.07)	0.76	2.06 (0.69–6.13)	0.2

*SBRT:* stereotactic body radiation therap, *BED*_10_: biologic effective dose, *ITV*: internal target volume, *PTV:* planning target volume, *D98:* minimum dose covering 98% of the target volume, *D99:* minimum dose covering 99% of the target volume, *PET:* positron emission tomography, *SUV:* standardized uptake value, *OR:* odds ratio, *CI:* confidence interval

### Dosimetric analysis

Median PTV volumes were 66.6 cc, 89.3 cc and 62 cc for the entire patient cohort, patients undergoing initial course of radiotherapy, and re-irradiation, respectively. The median PTV of initial radiation treatment group was significantly larger than that of re-irradiation group (*p* = 0.03). The median dose of hfSBRT was 65 Gy (range, 40–70 Gy) with BED_10_ of 107.25 Gy (range, 56–119 Gy) for the entire patient cohort. The majority of patients (66.7%) received ablative hfSBRT. There was no difference in median doses between the initial radiotherapy group (65 Gy and BED_10_ of 107.25 Gy) and re-irradiation group (64 Gy and BED_10_ 104.97 Gy) (*p* = 0.17).

Evaluation of OARs revealed a median lung V20 of 8.0% (IQR 4.5–17.2%) and a MLD of 11.5 Gy (IQR 6.7–16.8%). Median esophageal Dmax was 37.52 Gy (IQR 24.4–48.9 Gy), with a median esophageal V40 of 0 cc (IQR 0–0.6 cc). Median bronchial tree Dmax was 59.9 Gy (IQR 45.5–75.2 Gy), with a bronchial tree V50 was 0.96 cc (IQR 0–3.86 cc). The median great vessel Dmax was 68.2 Gy (IQR 53.5–76.6 Gy), and a median V50 of 0.94 cc (IQR 0.1–9.7 cc). Dosimetric parameters between initial radiotherapy treatment and re-irradiation groups were not statistically different apart from mean lung dose (MLD), which was higher in the initial radiotherapy treatment group (12.3 Gy vs. 9.9 Gy, *p* = 0.01) (Table 4).

**Table 4:** Dosimetric paraneters

Characteristic	Entire patient cohort (N = 78)	No prior in-field radiotherapy (N = 50)	Re-irradiation (N = 28)	*p*-value
PTV volume (cc): median (range)	66.6 (9.3–1201.8)	89.3 (9.3–1201.8)	62.0 (12.2–210.7)	0.03
Dose fractionation (total dose in Gy/fraction)				0.21
70/10	23 (29.5)	19	4	
65/10	22 (28.2)	12	10	
63/10	1 (1.3)	0	1	
60/10	6 (7.7)	3	3	
50/10	22 (28.2)	13	9	
42.5/10	1 (1.3)	1	0	
40/10	3 (3.8)	2	1	
Dose (Gy): median (range)	65.0 (40–70)	65.0 (40–70)	64.0 (40–70)	0.23
BED_10_ (Gy): median (range)	107.25 (56–119)	107.25 (56–119)	104.97 (50–119)	0.17
Total lung				
V5 (%): median (IQR)	24.0 (15.0, 41.7)	27.4 (16.7, 41.7)	19.3 (11.6, 36.3)	0.17
V20 (%): median (IQR)	8.0 (4.5, 17.2)	9.6 (5.2, 17.8)	7.8 (3.4, 11.0)	0.22
V40 (%): median (IQR)	3.2 (1.3, 5.8)	4.1 (1.5, 6.5)	2.3 (1.1, 5.1)	0.15
MLD (Gy): median (IQR)	11.5 (6.7,16.8)	12.3 (8.7, 18.7)	9.9 (4.7, 12.6)	0.01
Esophagus				
Dmax (Gy): median (IQR)	37.52 (24.4, 48.9)	41.6 (26.5, 52.9)	31.3 (21.3, 42.1)	0.09
V40 (cc): median (IQR)	0 (0, 0.6)	0 (0, 0.7)	0 (0, 0.01)	0.1
Primary bronchial tree/Trachea				
Dmax (Gy): median (IQR)	59.9 (45.5, 75.2)	55.6 (42.1, 77.3)	64.0 (49.8, 73.3)	0.81
V50 (cc): median (IQR)	0.96 (0, 3.86)	1.0 (0, 3.9)	1.0 (0, 3.2)	0.73
Great vessel				
Dmax (Gy): median (IQR)	68.2 (53.5, 76.6)	68.2 (47.1, 74.0)	69.2 (55.1, 77.5)	0.23
V50 (cc): median (IQR)	3.53 (0.1, 9.7)	3.2 (0, 13.1)	4.2 (0.8, 5.9)	0.18

*PTV:* planning target volume, *cc:* cubic centimeter, *Gy:* Gray, *BED*_10_: biologic effective dose, *V*_*x*_: volume of tissue exposed to x Gy or more, *Dmax:* maximum dose, *MLD:* mean lung dose, *IQR:* interquartile range

In total 28 (36%) individuals required re-irradiation. Seventeen (61%) underwent prior conventionally fractionated external beam radiation therapy (EBRT), 7 (25%) underwent prior SBRT, and 4 (14%) underwent both prior SBRT and EBRT. Prior EBRT doses ranged from 50 to 70.2 Gy, and prior SBRT doses ranged from 19.5 to 70 Gy (Table 1). Despite having received a prior course of radiotherapy, individuals undergoing re-irradiation received similar treatment as compared to individuals undergoing an initial radiotherapy course with respect to dose delivered and OAR dosimetric parameters (Table 4).

### Toxicity

Grade 2 or worse toxicity was observed in 28.2% of the patient population, including 22% (11/50) in the initial radiotherapy cohort and 39.3% (11/28) in the re-irradiation cohort (*p* = 0.10). The most common adverse event was pneumonitis accounting for 72.7% of events, of which 80% was grade 2 (Table 5). Only one case of ≥ 2 esophagitis was observed in an individual undergoing re-irradiation, which manifested as esophageal ulceration and stricture formation. The patient was managed conservatively with medication and a single esophageal dilation with good recovery. The esophagus received a Dmax of 57.8 Gy with a V40 of 6.3 cc during re-irradiation (Table 6).

**Table 5:** Late adverse events following ultracentral radiation therapy according to the CTCAE v 5.0

	All patient (n = 78)				No prior in-field radiotherapy (n = 50)				Patients with thoracic re-irradiation (n = 28)				*p*-value
	Grade 2	Grade 3	Grade 4	Grade 5	Grade 2	Grade 3	Grade 4	Grade 5	Grade 2	Grade 3	Grade 4	Grade 5	
Pneumonitis	12	3	–	–	6	1	–	–	6	2	–	–	0.12
Esophagitis	–	1	–	–	–	–	–	–	–	1	–	–	0.36
Airway toxicity	1	2	–	–	1	2	–	–	–	–	–	–	0.7
Hemorrhage	–	–	–	3*	–	–	–	1*	–	–	–	2*	0.13

All toxicity according to CTCAE v 5.0 scoring criteria

*The three patients who experienced hemoptysis with subsequent death were scored as having “possibly” treated related toxicity (one patient underwent bronchoscopy with clear progression of disease, one patient had radiographic evidence of progressive bilateral hilar disease just prior to death, and one patient cause of death was uncertain)

**Table 6:** Organ at risk dosimetry of patients with grade 3 or higher toxicity

	Prior irradiation	MLD (Gy)	TL V40 (%)	ESO Dmax (Gy)	ESO V40 (cc)	BT Dmax (Gy)	BT V50 (cc)	GV Dmax (Gy)	GV V50 (cc)	Reference constraint [10, 23]
Toxicity										
Pneumonitis										MLD ≤ 9 Gy
Patient 1	60 Gy/30fx	12.6	2							TL V40 ≤ 7%
Patient 2	70.2 Gy/39fx	6.3	2.2							
Patient 3	–	10.5	3.4							
Esophagitis										ESO Dmax ≤ 50 Gy
Patient 2	70.2 Gy/39fx			57.8	6.33					ESO V40 ≤ 1 cc
Airway toxicity										BT Dmax ≤ 60 Gy
Patient 4						73.6	7.7			BT V50 ≤ 1 cc
Patient 5						79.1	4.2			
Hemorrhage										BT Dmax ≤ 60 Gy
Patient 6	70 Gy/35fx					72.6	4.9	72.9	5.42	BT V50 ≤ 1 cc
Patient 7	50 Gy/5fx					77.4	5.7	73.7	16.8	GV Dmax ≤ 75 Gy
Patient 8	–					75	1.94	57.3	3.2	GV V50 ≤ 1 cc

*V*_*x*_: volume (percentage or cubic centimeters) of tissue exposed to x Gy or more, *Dmax:* maximum dose, *MLD:* mean lung dose, *TV:* total lung, *ESO:* esophagus, *BT:* bronchial tree, *GV:* great vessel, *fx:* treatment fractions, *Gy:* Gray

Three cases of airway toxicity were observed, all of which occurring in individuals undergoing an initial course of radiotherapy. The bronchial tree Dmax and V50 for the two grade 3 obstruction events were 73.6 Gy and 7.7 cc and 79.9 Gy and 4.2 cc, respectively. Both cases initially presented with a complete lobar consolidation, and patients underwent bronchoscopy with debulking of fibrotic and necrotic tissues, which resulted in complete resolution of the pulmonary consolidative process. The remaining grade 2 airway stenosis had a bronchial tree Dmax of 14.9 Gy and V50 of 0 cc and was managed with bronchodilators.

Lastly, three instances of grade 5 hemoptysis were observed, one in an individual undergoing an initial course of radiotherapy, and two in individuals undergoing re-irradiation. Their detailed dosimetric comparison with referent constraints in Table 6 indicated slightly higher V50, but not Dmax to great vessels. One hemoptysis event was scored as “possibly” related to treatment; two cases were deemed “unlikely” treatment related. In one case the patient underwent bronchoscopy with clear bleeding from progressive disease prior to death, and a second patient was seen to have clear radiographic evidence of progressive bilateral hilar disease prior to death.

No statistic differences were observed when comparing toxicity profiles between patients receiving initial radiotherapy treatment and re-irradiation (Table 5).

## Discussion

SBRT is a safe and effective treatment modality in the management medically inoperable tumors located in both the peripheral and central lung [1, 2, 10]. There continue to be questions surrounding the safety and efficacy in the management of ultracentrally located lung tumors given tumor proximity to critical OARs. Reasons for this include a diverse range of definitions for ultracentrally located lung tumors, variable dose and fraction regimens, and a lack of prospective trials reporting maximal tolerable doses to these high-risk regions. More published experiences in using SBRT for ultracentrally located lung tumors are needed to inform clinical practice. In the current study, we analyzed 78 consecutive patients treated with a hfSBRT course for ultracentral tumors. All patients underwent a 10-fraction course of treatment with doses ranging from 4 to 7 Gy per fraction. To date, this is the largest series for a patient population comprised exclusively of ultracentrally located tumors treated with a 10-fraction regimen.

### Outcomes

With 1- and 3-year LC rates of 83.5% and 65.4% respectively, the current investigation demonstrates favorable tumor control following a 10-fraction course of radiation therapy. A recent review by Chen et al. [16] detailing results from 10 ultracentral trials with a median BED_10_ of 78–103 Gy across all studies including a total of 250 patients reported a median 1-year and 2-year LC of 96% (range 63–100%) and 92% (57–100%). Our results did demonstrate inferior LC compared to this review. This is likely, at least in part, due to a significant percentage of our cohort undergoing re-irradiation, who had lower 3-year LC of 57.6% compared to 71.7% in the initial radiotherapy cohort. LC rates more closely approximate rates seen by Chen et al. when evaluating those undergoing an initial course of radiotherapy. The radioresistant nature of tumors having previously been irradiated may account for the numerically inferior LC seen in the re-irradiation group. Additionally, a BED_10_ ≥ 100 Gy is a well-established benchmark associated with not only superior LC, but OS [17]. Consistent with this, the two trials included in the review with the poorest LC (1-year LC of 70% and 63%) both utilized treatment regimens with a BED_10_ of 72 Gy or lower [18, 19]. BED_10_ in our cohort ranged from 56 to 119 Gy, with a majority of participants (66%) being treated with regimens with a BED_10_ ≥ 96 Gy. Only 5.1% of our cohort was treated to a BED_10_ less than 75 Gy, possibly impacting overall LC.

Comparison of OS and PFS in our patient population and the literature are difficult given the cohort diversity (33% primary intrathoracic disease, 29% recurrent disease, and 16% metastatic disease). When stratifying to primary, recurrent, or metastatic disease, our patient cohort does appear to fare as well or better than what has been previously reported. In the primary/recurrent setting Tekatli et al. [12] reported a 3-year OS of 20% in 47 patients undergoing a dose fractionation regimen of 5 Gy × 12 fractions (BED_10_ = 90 Gy), a value significantly lower than our 3-year rates of 71% and 52% for primary and recurrent disease, respectively. Li et al. [20] treated a total of 82 patients, including both centrally and peripherally located tumors, with primary or recurrent disease to a dose of 70 Gy in 10 fractions. In total 43 patients (52%) had centrally located tumors. In this trial, the overall survival was similar to our own cohort with a 2-year OS of 67%.

In the metastatic setting inferior survival outcomes has been demonstrated. Lischalk et al. [19] treated “high risk” metastatic central tumors, defined as tumor abutment or invasion of the mainstem bronchus, to a dose of 35–40 Gy in 5 fractions (BED_10_ = 60–72 Gy), reported a 1-year OS of 75%. Though not statistically significant (*p* = 0.16), there was a numerical difference seen in our cohort of patients when comparing OS for primary and metastatic disease with 3-year rates of 63% and 44%, respectively. The explanation for inferior survival is likely multifactorial including patients with poor performance status, more advanced disease, and prior progression through multiple systemic therapy regimens.

### Toxicity

Overall, our patient cohort experienced acceptable rates of treatment related toxicity. In all, 28% of individuals experienced ≥ grade 2 toxicity, including 12% with ≥ grade 3. Multiple trials treating ultracentrally located tumors have all reported ≥ grade 3 adverse events approximating 10% [17, 18, 21, 22].

Perhaps the most feared complication following SBRT for ultracentrally located thoracic tumors is a hemorrhagic event. In our own cohort we experienced three such cases; however, whether this was directly related to treatment or due to progression of disease is uncertain. These hemorrhagic events represented 100% of the grade 5 events. This appears consistent with the literature. Multiple studies have demonstrated low grade 4–5 events, and those that are seen are most commonly hemorrhagic events. Korztes et al. [22] and Horne et al. [21] both reported an isolated pulmonary hemorrhagic even representing ≤ 5% of their respective patient cohorts.

Tekatli et al. [12] reported a significantly higher rate of grade 5 toxicity, with 10 (21%) events, the majority of which (7/10) were hemorrhagic events. Important considerations from their study can be noted. In this cohort of 47 patients, a large proportion (n = 25, 53%) had endobronchial disease. Likewise, 50% (5/10) of those with treatment related mortality had endobronchial tumors. Similarly, the recently published phase II Hilus trial by Lindberg et al. [23] reported higher than expected treatment related toxicity. In this trial, 67 patients with ultracentrally located tumors were treated with a dose fractionation scheme of 7 Gy × 8 fractions prescribed to the 67% IDL. Patients were stratified by tumors located ≤ 1 cm from the main bronchi and trachea, and all others. In total, grade 5 events were seen in 10/65 (15%) of the patient population, 8 of which were secondary to bronchopulmonary hemorrhage. Univariate analysis demonstrated that the distance between the tumor and main bronchus was significant for fatal hemorrhage and grade 5 toxicity. Of note, a prescription to the 67% IDL corresponds to a “hot spot” of 150%. It is plausible that the combination of close tumor proximity to the main bronchus and the high maximum dose contributed to these higher rates of hemorrhage. Both trials stress the importance of proper patient selection, and careful treatment planning and evaluation, particularly in those in whom the highly vascularized bronchial wall may be compromised.

### Dosimetry

Little comparative dosimetric information for a 10-fraction course of central thoracic radiotherapy is available. The exception to this is the previously discussed work by Li et al. [20], of which 52% of the patient cohort had tumors that were located centrally. Overall, patients had a favorable toxicity profile, with pneumonitis and chest wall pain being the most frequently noted adverse events. A limited number of adverse events directly involving central or mediastinal structures were observed including 4 cases of ≥ grade 2 esophagitis, and one case of fatal hemoptysis. Based on these results, dosimetric constraints for a 10-fraction course of intrathoracic radiotherapy were proposed including an esophageal Dmax < 50 Gy and V40 < 1 cc, a bronchial tree/tracheal Dmax < 50 Gy and V50 < 1 cc, a great vessel Dmax ≤ 75 Gy and V50 < 1 cc, and a mean lung (MLD) ≤ 9 Gy with a total lung V40 ≤ 7% [20, 24].

In our own cohort, achieving many of these proposed OAR constraints proved difficult. For example, we observed a median primary bronchial tree/tracheal Dmax of 59.9 Gy and a median V50 of 0.96 cc implying approximately 50% of individuals exceeded the suggested constraint. Similarly, with a median great vessel Dmax of 62.2 Gy and median V50 of 3.53, constraints were not met in most cases. Despite this, the observed rate of adverse events directly involving these OARs was low. In our cohort, no adverse events involving the great vessels were observed. We observed a total of only five ≥ grade 3 adverse airway events, including the three previously discussed hemorrhagic events. Of those five, two had undergone prior radiation therapy, and all five exceeded both the suggested bronchial maximum and volumetric constraints proposed by Li et al. (Table 6). Our findings suggest that these proposed constraints may be conservative, and that these OARs can safely tolerate higher doses, though extreme caution should be taken, particularly in the setting of prior radiotherapy.

The currently enrolling phase I dose escalation SUNSET trial evaluating maximally tolerated dose for ultracentral NSCLC includes a dose level of 60 Gy in 10 fractions and includes corresponding dosimetric constraints [25]. These proposed constraints are largely more generous, particularly regarding volumetric constraints. For example, the primary bronchial tree/tracheal as well as the great vessel V60 is ≤ 10 cc. These more lenient constraints coupled with the lower treatment dose of 60 Gy should make appropriate OAR sparing more feasible without the sacrifice of tumor coverage. However, results from this trial are still pending, and caution utilizing this regimen should be utilized until its safety has been firmly established.

### Limitations

There are several limitations to this study apart from its retrospective nature. These include the relatively small patient cohort, and short follow up time, which is partly attributable to the high mortality rate in this patient population. Furthermore, though basic dosimetric parameters for individuals undergoing re-irradiation were gathered, a detailed dosimetric analysis was unable to be performed due to a limited availability of prior treatment plans.

Despite these limitations, the current investigation remains the largest series to detail the safety and efficacy of a 10-fraction course of radiotherapy for tumors located in a ultracentral location. Our inclusion of primary, metastatic and re-irradiation patients allows this study to provide data regarding efficacy of this treatment regimen in all 3 patient types commonly seen by radiation oncologists. Furthermore, this study demonstrates the safety profile of the 10-fraction treatment in both first course and re-irradiation patients.

## Conclusion

Risk-adapted hfSBRT to ultracentral lung tumors delivered with ablative dose over 10 fractions is a safe and effective treatment option, with acceptable rates of toxicity and good rates of tumor control.

## Data Availability

Study data underlying this article will be shared on reasonable request to the corresponding author.

## Abbreviations

hfSBRT: Hypofractionated stereotactic body radiotherapy
OS: Overall survival
PFS: Progression free survival
LC: Local control
OAR: Organ at risk
Dmax: Maximum dose
SBRT: Stereotactic body radiotherapy
NSCLC: Non-small cell lung cancer
GTV: Gross tumor volume
PTV: Planning target volume
BED: Biologic effective dose
IDL: Isodose line
4D: 4-Dimensional
CT: Computed tomography
ITV: Internal target volume
MaxIP: Maximum intensity projection
PET: Positron emission tomography
MLD: Mean lung dose
KM: Kaplan–Meier
EBRT: External beam radiotherapy
